# *Prevotella histicola* Mitigated Estrogen Deficiency-Induced Depression *via* Gut Microbiota-Dependent Modulation of Inflammation in Ovariectomized Mice

**DOI:** 10.3389/fnut.2021.805465

**Published:** 2022-01-26

**Authors:** Furong Huang, Xiujie Liu, Sheng Xu, Sitao Hu, Sisi Wang, Dibang Shi, Kaicheng Wang, Zhongxiang Wang, Qiongqiong Lin, Shan Li, Siyuan Zhao, KeKe Jin, Chuang Wang, Lei Chen, Fangyan Wang

**Affiliations:** ^1^Department of Orthopaedics, The First Affiliated Hospital of Wenzhou Medical University, Wenzhou, China; ^2^Key Laboratory of Intelligent Critical Care and Life Support Research of Zhejiang Province, Wenzhou, China; ^3^School of Basic Medical Sciences, Wenzhou Medical University, Wenzhou, China; ^4^The Second Affiliated Hospital and Yuying Children's Hospital of Wenzhou Medical University, Wenzhou, China; ^5^Department of Pharmacology, Provincial Key Laboratory of Pathophysiology in Ningbo University School of Medicine, Ningbo, China

**Keywords:** *Prevotella histicola*, inflammation, depression, BDNF, microbiota, tight junction protein

## Abstract

**Background:**

Estrogen deficiency-induced depression is closely associated with an imbalance in intestinal microbiota and inflammation. *Prevotella histicola* (*P. histicola*), an emerging probiotic, apparently improves inflammatory responses. This study aims to verify the antidepressant-like effects of *P. histicola* and clarify its potential mechanisms.

**Methods:**

Mice were treated with *P. histicola* and cohousing after ovariectomy (OVX). The changes in depression-like behaviors among mice were examined by behavioral tasks, and alterations in the microbiota were detected through 16S rRNA sequencing. Changes in neuronal injury, protein synthesis, inflammatory factors, intestinal permeability, and nerve proliferation were observed by H&E, Nissl staining, qRT-PCR, western blotting, and immunofluorescence.

**Results:**

*P. histicola* significantly reduces depression-like behaviors and neuronal damage induced by estrogen deficiency. Additionally, *P. histicola* significantly increases the abundance of intestinal flora, especially *Lactobacillus* and *Akkermansia*. Meanwhile, the cohoused mice also had a better emotional state and neutral structure compared with OVX mice. *P. histicola* was also found to upregulate tight junction proteins ZO-1, occludin, claudin-1, and MUC2 in the ileum and colon and reduce the levels of inflammatory factors VCAM, MCP-1, IL-6, IL-8, and TNF-α, mainly in the ileum, colon, and decrease the expression of COX-2, TLR4, Myd88, JNK, MCP-1, IL-6, IL-8, and TNF-α in the hippocampus. Moreover, significant downregulation of apoptosis (caspase-3 and caspase-8) and upregulation of neurotrophic factors (BDNF and Ki-67) were observed after *P. histicola* treatment.

**Conclusion:**

Our data show that *P. histicola* significantly mitigates depression of OVX mice through improvement in intestinal microbiota to repair intestinal leakage and inhibit central inflammation to promote the expression of BDNF for hippocampal neurogenesis. *P. histicola* may be therapeutically beneficial for PMD.

## Introduction

Menopause, characterized by the permanent cessation of menstruation, is associated with a series of diseases such as osteoporosis, cardiovascular disease, and breast cancer (1–3). In addition to physical symptoms, many patients suffer from a wide range of psychological impairments, especially depressive disorders, which can aggravate physical injuries. Depression is considered among the most serious diseases in the 21 st century and is listed as the second major cause of disability among humans (4). Estrogen therapy is currently the main treatment for postmenopausal depression (PMD), which can significantly reduce related mental problems (5). However, the long-term use of estrogen is limited, as it may increase the risk of breast cancer, ovarian cancer, stroke, and cardiovascular diseases (6, 7). Therefore, there is an urgent need for an optimal alternative form of therapy with fewer side effects.

In recent years, the role of intestinal microbiota in the “gut–brain” has been increasingly recognized (8) and has been shown to be closely related to depression (9, 10). Previous related studies suggest that dysbiosis can disrupt the intestinal mucosal barrier (11), hypothalamic–pituitary–adrenal axis (12) and neurogenesis (13) to aggravate depression. Although the involvement of gut microbes in the pathogenesis of depression has been well-documented, studies on microbiota in PMD are still in the primary stage. Based on a recent study that reported significant changes in the intestinal flora with hypoestrogenic, Fuhrman et al. (14) found that postmenopausal women with elevated urinary ratio of hydroxylated estrogen metabolites to parent estrogen showed a more diverse gut microbiome. The data of Li et al. (15) showed that the relative abundance of *Cyanobacteria* which is related to toxin (16) was higher in the ovariectomy (OVX) group compared with the sham group. Therefore, dysbiosis might be a pathogenic factor that exacerbates estrogen deficiency depression.

Neuroinflammation is among the most significant contributing risk factors of depression (17). Proinflammatory cytokines such as interleukin (IL)-1β, IL-8, and tumor necrosis factor-α (TNF-α) among patients with depression have been reported to be significantly upregulated (18, 19). The dysfunction of the intestinal mucosal barrier induced by dysbiosis is involved in the proinflammatory response in depression (20), suggesting that dysbiosis-induced gut leakage contributes to neuroinflammation in depressed animals (21). In addition to neuroinflammation, brain-derived neurotrophic factor (BDNF), an important member of the neurotrophic family, plays a critical role in the regulation of synaptic plasticity and the expression level of monoamine neurotransmitters (22–24). Increasing evidences show that BDNF exerts an antidepressive effect mainly by promoting neuronal proliferation (25). The decreased level of hippocampal BDNF in a rat model of depression was found to inhibit neuronal proliferation (26). Moreover, central inflammation was associated with reduced BDNF, as confirmed by the series of studies on animals and humans (27, 28). Systemic administration of proinflammatory cytokines IL-1β or LPS induced a notable reduction in hippocampal BDNF content in depressive rats (29, 30), with similar results that found in depressive patients exhibiting a decrease in neuroinflammation-induced BDNF (31). Intestinal microbiota studies indicate that dysbiosis may be a contributing factor to the decreased expression of BDNF among depressive mice through inflammatory activation caused by dysbiosis-induced gut leakage (32, 33).

Moreover, a recent OVX animal study showed that ovarian progesterone reduced depression-like behaviors driven by intestinal flora disorder (34). Therefore, it is worth seeking effective methods of PMD therapy based on intestinal microbiota optimization. *Prevotella histicola* (*P. histicola*), a gram-negative obligate anaerobic bacterium of the *Prevotella* genus, was shown to downregulate the proinflammatory cytokine interferon-γ (IFN-γ) and IL-17, reducing the activation of microglia and astrocytes in a model of multiple sclerosis (35). Our recent study showed that the oral application of *P. histicola* significantly attenuated osteoporosis in an OVX mouse model (1). However, it is not clear whether *P. histicola* can mitigate estrogen deficiency-induced depression.

This study explores whether *P. histicola* mitigates estrogen deficiency-induced depression dependent on microbiota-mediated neuroinflammation and neuroprotective effects among OVX mice. Our findings provide a new therapeutic target and an entry point for understanding the biological mechanisms of PMD.

## Materials and Methods

### Animals

All experimental procedures were approved by the Experimental Animal Center of Wenzhou Medical University. Female C57BL/6 mice weighing 20–22 g at 10–12 weeks of age were purchased from Beijing Weitonglihua Experimental Animal Technology Co. Ltd. (Beijing, China). The mice were contained at a constant temperature (22°C ± 2°C), maintained a 12-h light/dark cycle, and allowed free access to food and water. All mice were acclimated for 1 week before the experiment. All experiments were conducted according to the Animal Health Care and Use Guidelines of the Wenzhou Medical University.

### Incubation Culture of *P. histicola*

Anaerobic *P. histicola* (strain number: DSM19854) from Deutsche Sammlung von Mikroorganismen und Zellkulturen (DSMZ) was cultured in modified PYG medium (the media component is shown in Supplementary Table 1) at 37°C for 24 h under anaerobic conditions.

### Ovariectomy

The mice were randomly divided into Sham, OVX, OVX + *P. histicola*, and OVX + cohousing (*n* = 7–8/group) groups. After disinfection with 10% povidone iodine, the skin was exposed to aseptic surgery under anesthesia with an intraperitoneal injection of 4% chloral hydrate (10 mg/kg). An abdominal incision through the skin and muscle of the abdomen was made to remove the bilateral ovaries. In the sham group, similar to the experimental groups, incision to gain access to the abdominal cavity was made. However, only the corresponding volume of fat around the ovary was removed. All the mice were injected with penicillin 50,000 U/D for 3 days and were left to recover for 1 week.

### Experimental Design

**Figure 1:**
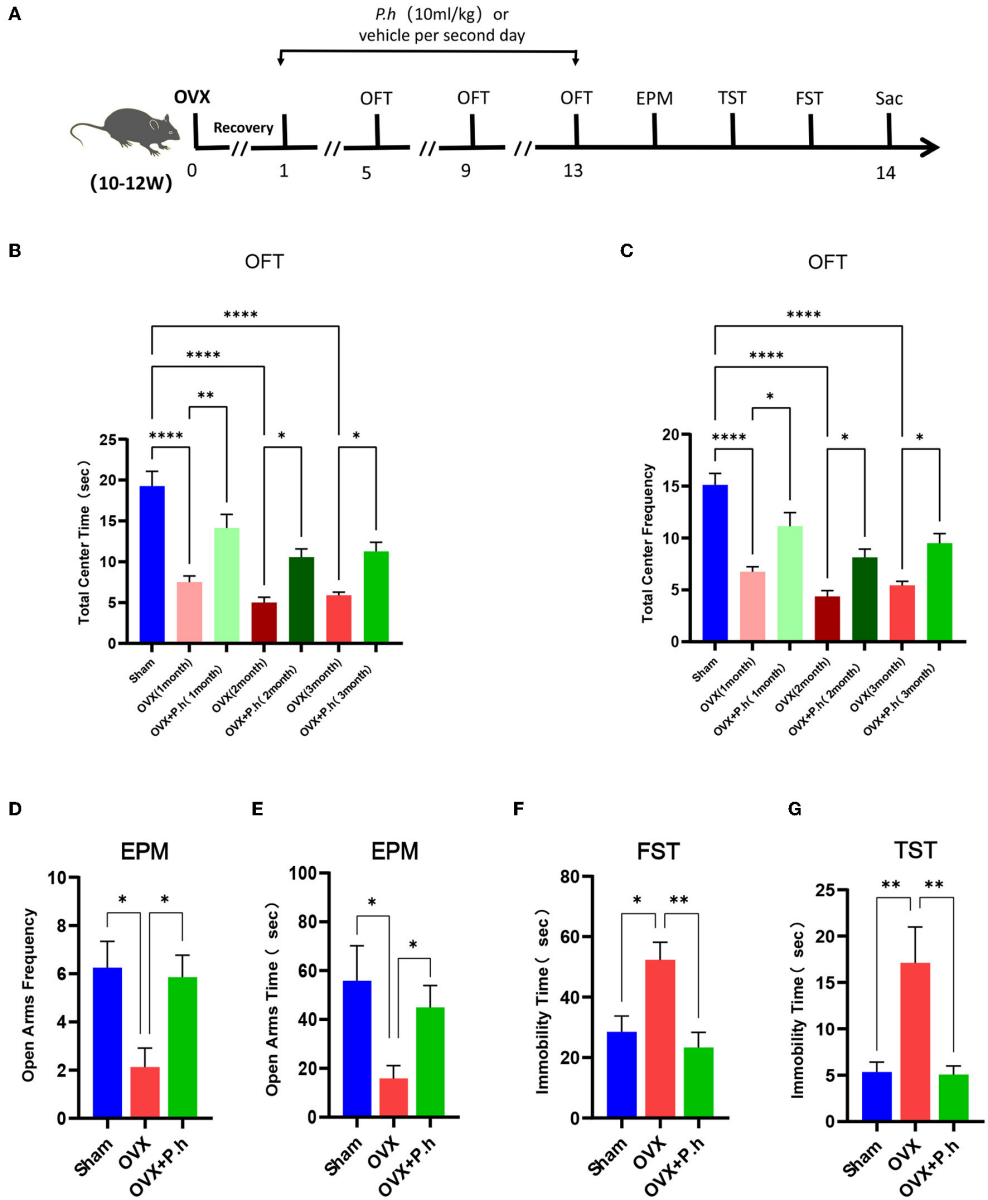
*P. histicola* alleviated the depression-like behaviors in OVX mice. The mice were ovariectomized or sham operated and then given 1 × 109 CFU *P. histicola* medium per 2 days for 12 weeks. The sham group and OVX group were given the same volume of sterile medium. The depression-like behavior was measured by OFT, EPM, TST, and FST. **(A)** Schematic diagram of the experimental procedures. **(B)** Central area time of OFT. **(C)** Transition times of OFT. **(D)** Total time in open arms of EPM. **(E)** Transition times in open arms of EPM. **(F)** Immobility time of TST. **(G)**. Immobility time of FST. Data are presented as means ± SEM (*n* = 7–8/group). ^*^p < 0.05, ^**^p < 0.01, ^****^p < 0.0001.

The open field test (OFT) was conducted every 4 weeks after the operation, and the mice were exposed to a series of behavioral tests and sacrificed 12 weeks later (Figure 1A). To reduce the stress response, the mice were acclimated for at least 30 min after being transported to the laboratory. The experimental apparatus was wiped with 75% ethanol solution before each behavioral test. After all the experiments, the mice were anesthetized with chloral hydrate, after which plasma and tissues were collected for subsequent investigation. The mice were further euthanized upon exhibiting a 20% weight loss or debilitating signs, such as arched back, less movement, and head shrinkage.

### Cohousing

Three treated and three untreated OVX mice were cohoused in a single cage and maintained for 12 weeks (three cages in total, *n* = 18).

### Behavioral Tests

#### Open Field Test

The experiment was conducted in a square box (40 cm × 40 cm × 40 cm). The floor was further divided into the central (15 cm × 15 cm) and surrounding areas. All the mice were placed in the surrounding area and allowed to move freely for five min. The time spent in the central area and the frequency of entering the central area were used as indicators to evaluate the degree of depression.

#### Elevated Plus-Maze Test

An elevated plus-maze (length, 35 cm; width, 5 cm; closing arm height, 20 cm; ground height, 61 cm) was used for this test. Each mouse was placed at the end of the closed arm, with the head facing the center. The time and frequency of the mouse entering the open arm within five min were recorded only when the mouse extended all its limbs into any given arm.

#### Forced Swimming Test

The forced swimming test (FST) is also a behavior despair-based test. Each mouse was placed in a glass cylinder (diameter, 20 cm; height, 50 cm) containing water at a depth of 30 cm (25°C) and trained to swim for 15 min. After 24 h, the mouse was subjected to a 5-min forced swimming test, during which the immobility time was recorded.

#### Tail Suspension Test

Under dim light conditions (~40 lx), the mice were suspended using duct tape from a metal bar 1 cm from the start of the tail. The behavior of the mice was recorded using a video camera for five min.

### Gut Microbiota Analysis

Mice fecal samples collected at the end of the animal experiments were frozen at −80°C for storage. The gut microbiota was detected using 16S ribosomal ribonucleic acid (16S rRNA) sequencing. Amplification of the 16S rRNA gene extracted from fecal samples was performed using the Illumina MiSeq platform. The alpha diversity index of the intestinal flora was analyzed using Ace, Chao. Principal component analysis (PCA) plots for visualization of UniFrac dissimilarity were generated using the QIIME pipeline. The characteristics of differences were assessed through linear discriminant analysis effect size (LEfSe).

### Quantitative Real-Time PCR

Total RNA from hippocampal and intestinal tissues was extracted using TRIzol (Yamei, Shanghai, China). The isolated RNA was reverse-transcribed to cDNA using a kit (Vazyme, Nanjing, China). The cDNA obtained was subjected to PCR using primers designed for interleukin-6 (IL-6), interleukin-8 (IL-8), IL-17, tumor necrosis factor-α (TNF-α), macrophage chemoattractant protein 1 (MCP-1), cysteinyl aspartate-specific proteinase 3 (caspase-3), cysteinyl aspartate-specific proteinase 8 (caspase-8), BDNF, zonula occludens-1 (ZO-1), occludin, claudin-1, recombinant mucin 2 (MUC2), 5-hydroxytryptamine receptor 1A (5-HT_1A_), 5-hydroxytryptamine receptor 1 B (5-HT_1B_), dopamine receptor 1 (DA-1), dopamine receptor 2 (DA-2), and β-actin (primer sequences are listed in Supplementary Table 2). Gene expression was determined using the SYBR Green kit (Vazyme, Nanjing, China). All the results were normalized against β-actin expression using the Thermal Cycler Dice Real Time system (TaKaRa Company, Japan).

### Western Blotting Analysis

Total protein samples were extracted by treating tissues with RIPA lysis buffer (Biosharp, China). Protein concentration was determined using a Pierce BCA protein detection kit (Beyotime Institute of Biotechnology, Shanghai, China). The protein was separated using 10% SDS-PAGE and transferred to a polyvinylidene fluoride membrane. After blocking in 5% skimmed milk, it was incubated with the primary antibodies occludin (Proteintech, 27260-1-AP, 1:1000), cyclooxygenase-2 (COX-2) (Proteintech, 66351-1-Ig, 1:1000), BDNF (Proteintech, 28205-1-AP, 1:1000), vascular cell adhesion molecule (VCAM) (Huabio, SA05-04, 1:1000), TLR4 (Bioworld, BS3489, 1:1000), Myd88 (Bioworld, BS3521, 1:1000), phospho c-Jun N-terminal kinase (p-JNK) (Proteintech, 80024-1-RR, 1:1000), JNK (Proteintech, 24164-1-AP, 1:1000), caspase-3 (Cell Signaling Technology, 9662S, 1:1000), and β-Tubulin (Proteintech, 10068-1-AP, 1: 50,000) at 4°C overnight. After washing with TBST three times, the bands were incubated with appropriate antirabbit or antimouse secondary antibodies at room temperature for 1 h. The imprinting was observed *via* chemiluminescence (Pierce) and an Odyssey imaging system (Li-Cor-Biosciences, NE).

### Immunofluorescent Staining

Dewaxed paraffin-embedded sections were hydrated, washed in PBS for 5 min, and placed in boiling citric acid buffer (0.01 mol/L, pH 6.0) to repair the antigens. The sections were incubated with primary antibodies against Iba1 (Novus, NB100-1028, 1:500), ZO-1 (Proteintech, 66378-1-IG, 1:100), Occludin (Proteintech, 27260-1-AP, 1:100), CD68 (Proteintech, 28058-1-AP, 1:100), and Ki-67 (Affinity, AF0198, 1:100) at 4°C overnight. After washing with PBS, the sections were incubated with the secondary antibody at room temperature for 1 hour and washed with PBS. The nuclei were stained with 4', 6-diamino-2-phenylindole (Beyotime Biotechnology, China). Immunofluorescence images were taken using a fluorescence microscope (Olympus, Japan) and analyzed using ImageJ software (National Institute of Mental Health, Bethesda, MA, USA) to obtain the mean fluorescence intensity.

### Nissl Staining

Widely performed to identify neuronal injury, Nissl staining was used to detect Nissl bodies in neuronal cytoplasm and dendrites, which was conducted according to the conventional method in instruction (Solarbio, Beijing, China). The hippocampus was observed carefully using an optical microscope (Olympus, Tokyo, Japan). The results were quantified as (Nissl-positive cells)/(total cells) × 100%.

### Statistical Analysis

All statistical analyses were performed using SPSS version 19.0 (IBM, Armonk, NY, USA). Data are expressed as mean ± SEM. The expression of occludin in the ileum was analyzed using an unpaired *t*-test, and the total center time and frequency in the OFT were analyzed using a two-way ANOVA with Bonferroni's multiple comparisons *post hoc* test. The rest of the statistical analysis was applied for one-way ANOVA tests. Differences were considered statistically significant at *p* < 0.05.

## Results

### *P. histicola* Alleviated Depressive Behaviors Caused by Estrogen Deficiency

The OVX mice showed significant uterine atrophy, indicating that their ovaries had been successfully removed (Supplementary Figure 1A). Meanwhile, our results found that the mice continued to gain weight after OVX, and *P. histicola* had no significant effect on the weight of OVX mice (Supplementary Figure 1B). To investigate the effects of *P. histicola* on estrogen deficiency-induced depression, a series of behavioral tests including the EPM, OFT, TST, and FST were conducted. EPM and OFT are used to evaluate the exploratory behaviors of mice and ascertain their depression or anxiety levels. Our experimental results showed that mice in the OVX group had significantly reduced time and frequency to enter the open arm and also the central area compared with those in the sham group (*p* < 0.05, Figures 1B–E). However, these effects were significantly alleviated with the intervention with *P. histicola* (Figures 1B–E). Correspondingly, the total distance and speed of OVX mice in OFT were also significantly increased (Supplementary Figures 2A,B). To evaluate desperate behaviors, TST and FST were adopted. The extension of immobility time indicated the weakening of the survival consciousness of animals. Our data showed that the increased immobility time of OVX mice in the TST and FST was significantly reduced after *P. histicola* treatment, indicating that the survival consciousness of mice was improved by *P. histicola* (*p* < 0.05, Figures 1F,G). These results suggest that *P. histicola* treatment effectively improves estrogen deficiency-induced depressive behaviors in mice.

### *P. histicola* Reduced the Hippocampal Neuronal Damage Caused by Estrogen Deficiency

**Figure 2:**
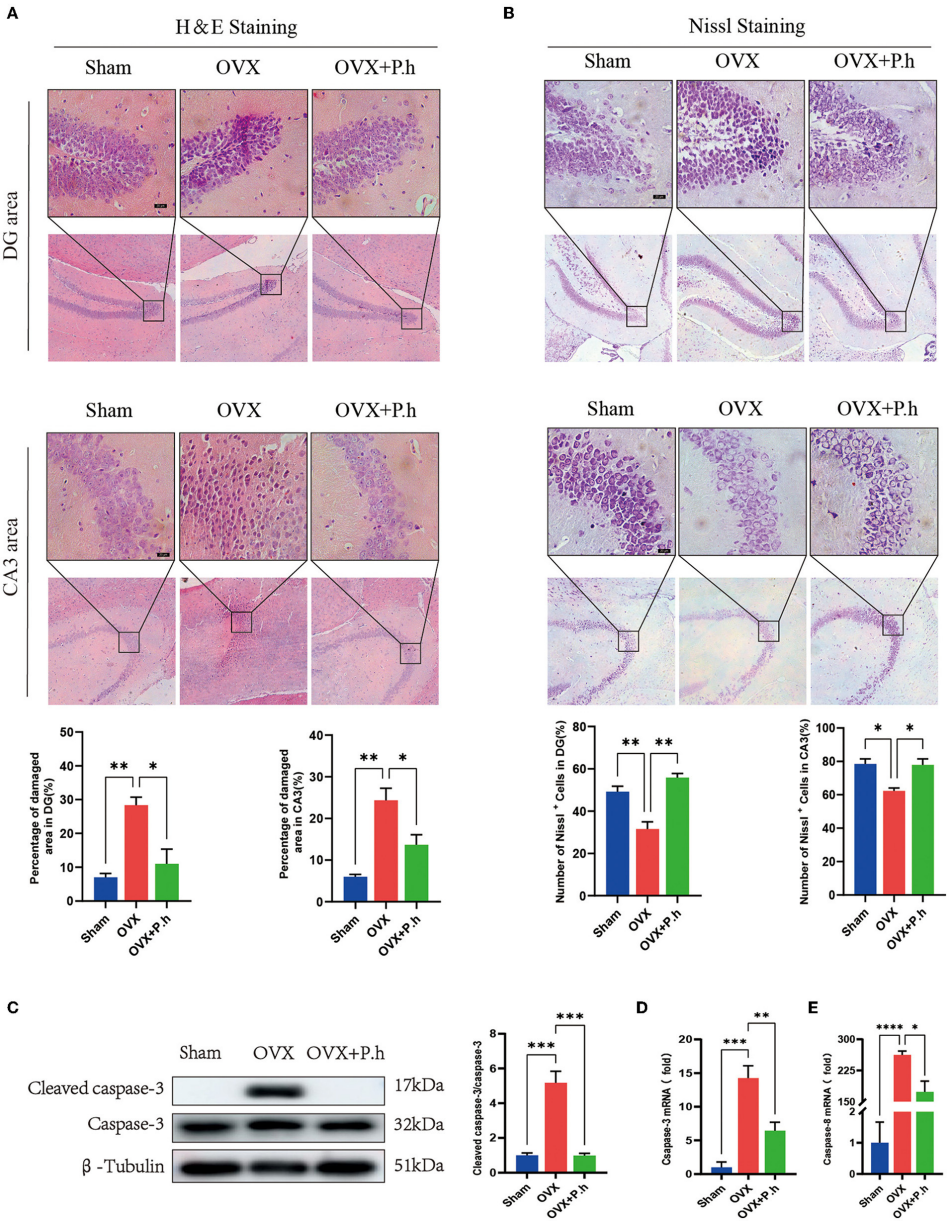
*P. histicola* reduced hippocampal injury and neuronal apoptosis in OVX mice. **(A)** H & E staining and its quantifications. Magnification 100 × and 400 ×. Scale bar = 20 μm. **(B)** Nissl staining and its quantifications. Magnification 100 × and 400 ×. Scale bar = 20 μm. **(C)** Cleaved caspase-3 protein level and the ratio of cleaved caspase-3 to caspase-3 in hippocampus. **(D,E)**. The mRNA expression of caspase-3 **(C)** and caspase-8 **(D)** detected by qRT-PCR. Data are presented as means ± SEM (*n* = 4/group). ^*^p < 0.05, ^**^p < 0.01, ^***^p < 0.001, ^****^p < 0.0001.

Since pathological damage to neurons was the key link of depression, H&E and Nissl staining of the hippocampus were performed to evaluate the effects of *P. histicola* treatment on estrogen deficiency-induced neuronal structure injury. As shown by H&E staining, pyknosis was observed in the nuclei of dentate gyrus (DG) granule cells and hippocampal CA3 pyramidal neurons in OVX mice, which was attenuated by *P. histicola* treatment (*p* < 0.05, Figure 2A). Nissl staining showed that treatment with *P. histicola* restored the reduction of Nissl bodies in OVX mice (*p* < 0.05, Figure 2B). Moreover, the expression of caspase-3 and caspase-8 was also decreased after *P. histicola* treatment in OVX mice (*p* < 0.05, Figures 2C–E). These data indicate that *P. histicola* prevents estrogen deficiency-induced neuronal structure injury and promotes neuronal protein synthesis in mice.

### *P. histicola* Recovered the Intestinal Flora Disorder Caused by Estrogen Deficiency

**Figure 3:**
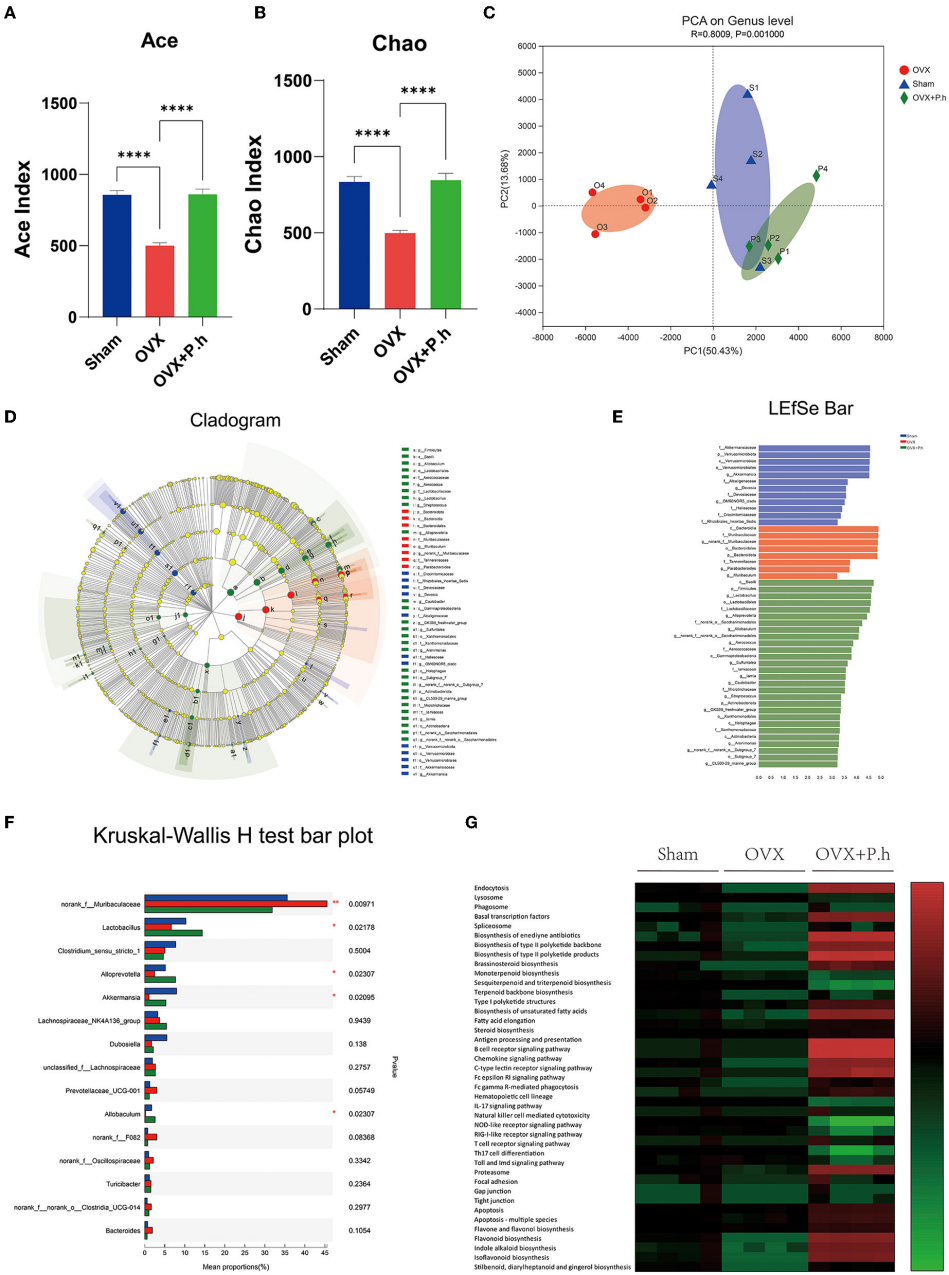
*P. histicola* restored the intestinal flora disorder of OVX mice. **(A,B)** Alpha diversity index ACE **(A)** and Chao **(B)**. **(C)** PCA on gene level of beta diversity analysis. **(D,E)** LDA effect size analysis and LDA discrimination results of each level. **(F)** Intergroup difference test at genus level. **(G)** The KEGG metabolic pathways of PICRUSt2. Data are presented as means ± SEM (*n* = 4/group). ^****^p < 0.0001.

The microbiota was evaluated using 16S rRNA sequencing and bioinformatics analysis. The Ace and Chao analysis showed a significant reduction in the abundance of intestinal flora in OVX mice, which was reversed by gavage of *P. histicola* (*p* < 0.0001, Figures 3A,B). However, there were no differences in the Shannon and Simpson indices among the three groups (Supplementary Figures 3A,B), indicating that the OVX and *P. histicola* treatment affected the abundance of intestinal flora rather than diversity. As shown in the PCA analysis, the intestinal flora of OVX mice showed a noticeable change compared with the sham group. However, *P. histicola* reversed this microbiome disorder (Figure 3C). Different biomarkers between groups were compared using LEfSe (Figures 3D,E). It is worth noting that OVX mice showed a decrease in *Lactobacillus, Alloprevotella, Akkermansia, and Allobaculum* and an increase in the abundance of *Muribaculaceae*, which were reversed by *P. histicola* (Figure 3F). The KEGG pathway of PICRUSt2 demonstrated distinct differences in transport and catabolism, the metabolism of terpenoids and polyketides, immune system, tight junction, cell growth and death, and biosynthesis of other secondary metabolites (*p* < 0.05, Figure 3G), suggesting that *P. histicola* might influence these pathways by regulating the microbiome to mitigate estrogen deficiency-induced depression.

### *P. histicola* Reduced the Inflammation of the Intestinal Wall Caused by Estrogen Deficiency

**Figure 4:**
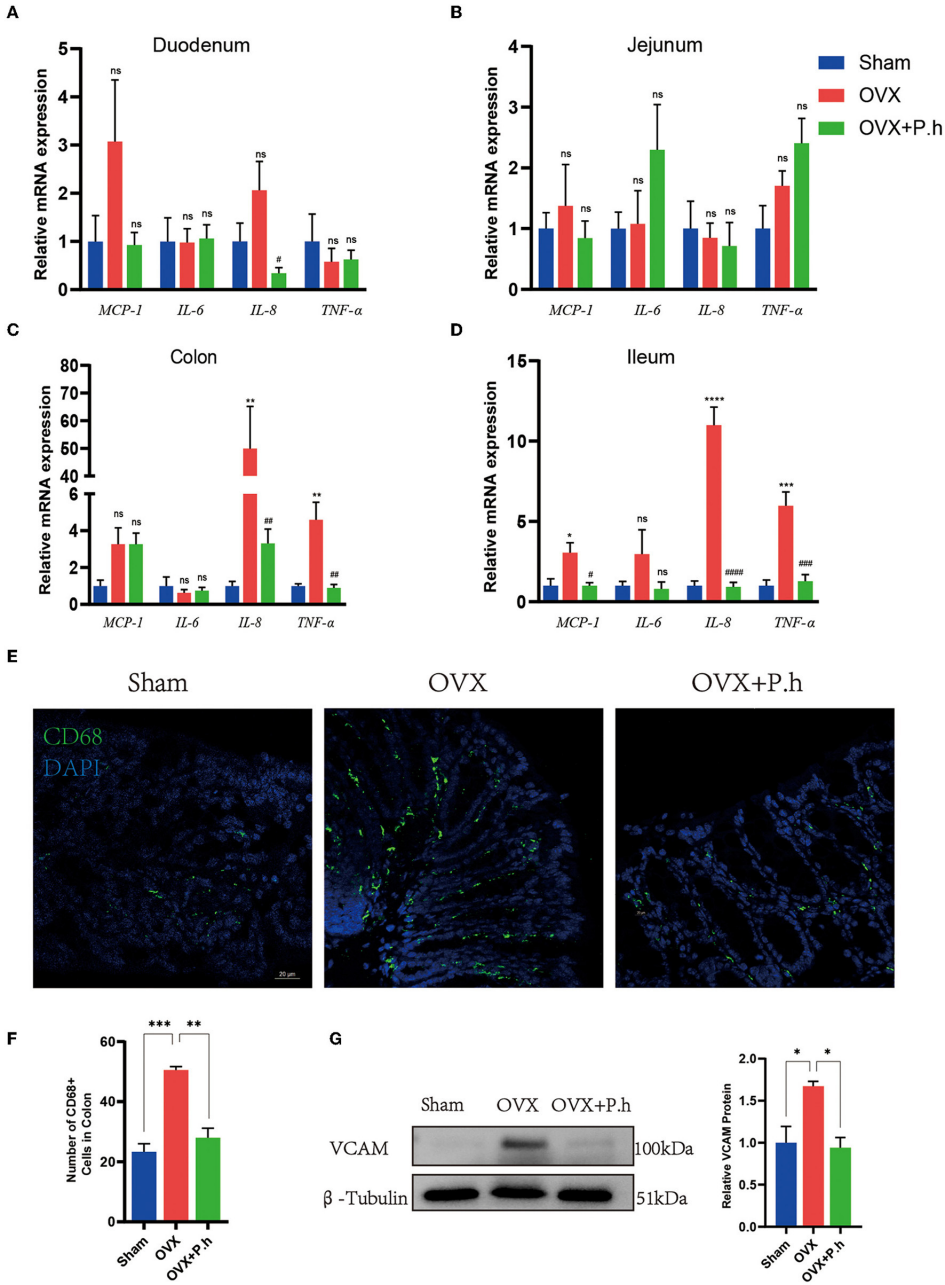
*P. histicola* inhibited the activation of intestinal macrophage and downregulated the inflammation factors level in OVX mice. **(A)** The mRNA expression of MCP-1, IL-6, IL-8, TNF-α in duodenum. **(B)** The mRNA expression of MCP-1, IL-6, IL-8, TNF-α in jejunum. **(C)** The mRNA expression of MCP-1, IL-6, IL-8, TNF-α in ileum. **(D)** The mRNA expression of MCP-1, IL-6, IL-8, TNF-α in colon. **(E,F)** Immunofluorescence **(E)** and quantification **(F)** of colon macrophage marker CD68. Magnification 400 ×. Scale bar = 20 μm. **(G)** VCAM protein level and the ratio of VCAM to β-Tubulin in colon. Data are presented as means ± SEM (*n* = 3–4/group). ^*^p < 0.05 vs. sham, ^**^p < 0.01 vs. sham, ^***^p < 0.001 vs. sham, ^#^*p* < 0.01 vs. OVX, ^*##*^*p* < 0.01 vs. OVX, ^*###*^*p* < 0.001 vs. OVX, ^*####*^*p* < 0.0001 vs. OVX, ns *p* > 0.05, ^****^p < 0.0001.

Intestinal inflammation resulting from disrupted intestinal microbiota is critical for systemic inflammation. Therefore, we employed qRT-PCR, western blotting, and immunofluorescence to analyze the proinflammatory factors MCP-1, IL-6, IL-8, TNF-α, adhesion molecule VCAM, and macrophage marker CD68. Compared with the sham group, the expression of ileal and colonic inflammation factors IL-8 and TNF-α and the level of MCP-1 in the ileum were significantly increased in OVX mice, which were downregulated by *P. histicola* treatment. *P. histicola* also reduced IL-8 levels in the duodenum significantly (Figures 4A–D). Immunofluorescence and western blotting showed that the expression of the colon macrophage marker CD68 and macrophage adhesion factor VCAM protein in OVX mice were significantly higher than those in the sham group, and *P. histicola* downregulated the levels of VCAM and CD68 (*p* < 0.05, Figures 4E–G). These results suggest that *P. histicola* reversed the activation of intestinal macrophages, hence inhibiting the release of inflammatory factors caused by estrogen deficiency.

### *P. histicola* Improved Intestinal Permeability Caused by Estrogen Deficiency

**Figure 5:**
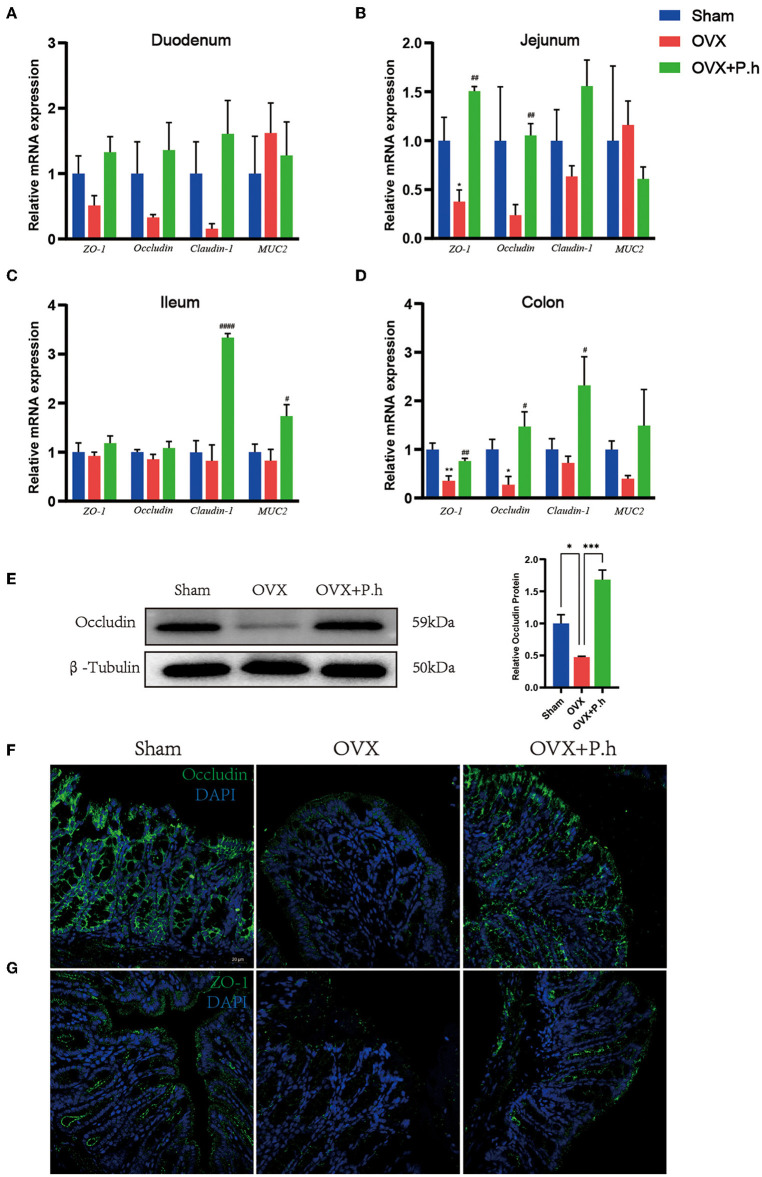
*P. histicola* restored intestinal leakage in OVX mice. **(A)** The mRNA expression of ZO-1, occludin, claudin-1, MUC2 in duodenum. **(B)** The mRNA expression of ZO-1, occludin, claudin-1, MUC2 in the mRNA expression of ZO-1, occludin, claudin-1, MUC2 in jejunum. **(C)** The mRNA expression of ZO-1, occludin, claudin-1, MUC2 in ileum. **(D)** The mRNA expression of ZO-1, occludin, claudin-1, MUC2 in colon. **(E)** Western blotting analysis of occludin in colon. **(F)** Immunofluorescence of occludin in colon. Magnification 400 ×. Scale bar = 20 μm. **(G)** Immunofluorescence of ZO-1 in colon. Magnification 400 ×. Scale bar = 20 μm. Data are presented as means ± SEM (*n* = 3–4/group). ^*^ *p* < 0.05 vs. sham, ^**^ *p* < 0.01 vs. sham, ^***^ *p* < 0.001 vs. sham, ^#^*p* < 0.01 vs. OVX, ^*##*^*p* < 0.01 vs. OVX, ^*####*^*p* < 0.0001 vs. OVX.

The increase in intestinal permeability played a key role in the upregulation of inflammatory level. To evaluate the effects of *P. histicola* treatment on estrogen deficiency-induced gut leakage, H&E was used to analyze the structure of the intestine. qRT-PCR, western blotting, and immunofluorescence were used to detect the expression of tight junction protein ZO-1, occludin, claudin-1, and MUC2. H&E staining showed no typical histological damage to the intestinal wall structure in OVX mice (Supplementary Figure 4A). However, the results of the qRT-PCR indicated that *P. histicola* significantly increased the levels of ZO-1 and occludin in the jejunum, claudin-1 and MUC2 in the ileum, and ZO-1, occludin, and claudin-1 in the colon (Figures 5A–D). Immunofluorescence and western blotting (Figures 5E–G) also confirmed the qRT-PCR data, indicating that *P. histicola* mitigated intestinal leakage in OVX mice.

### *P. histicola* Reduced Hippocampal Inflammation Caused by Estrogen Deficiency

**Figure 6:**
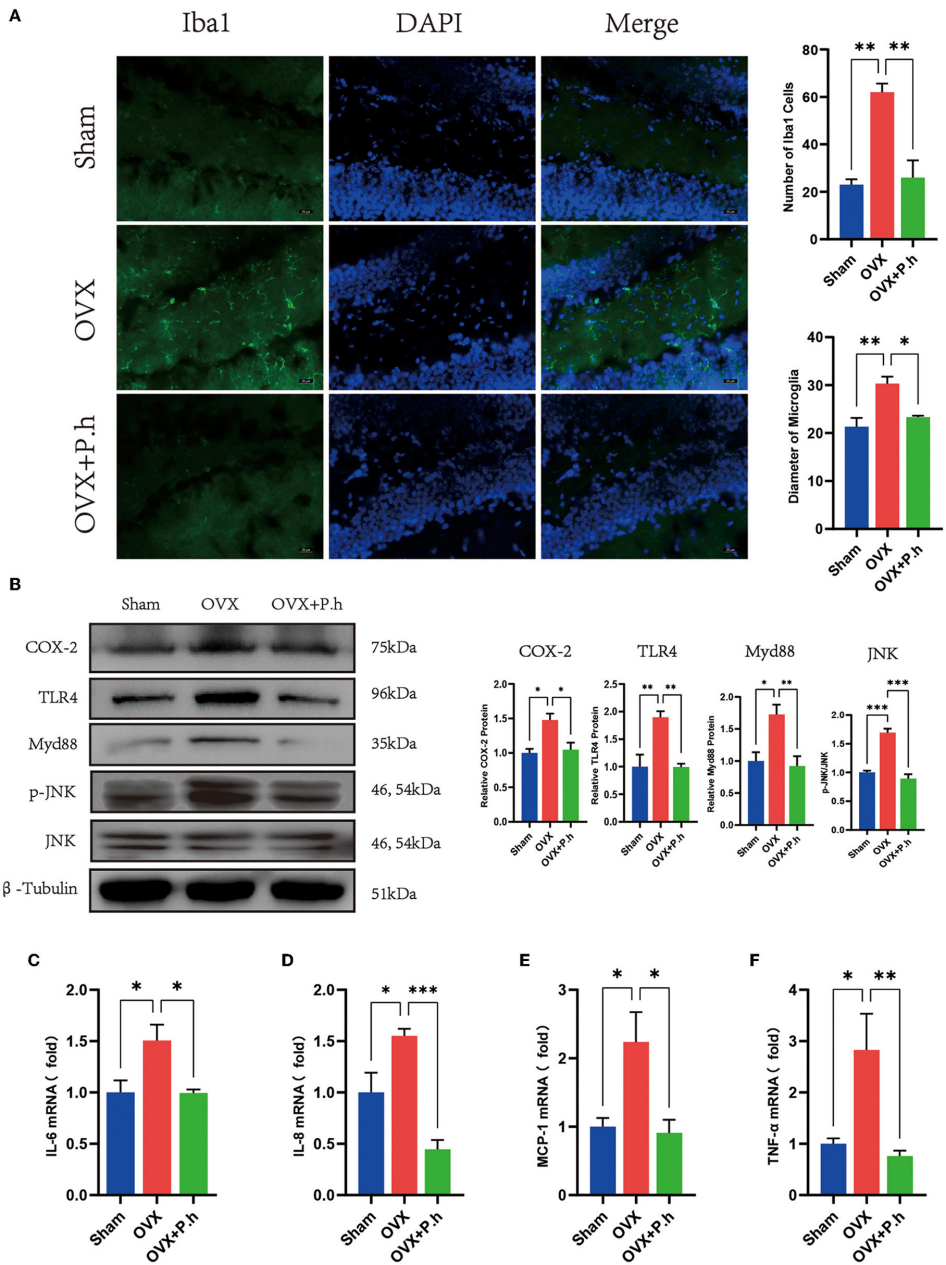
*P. histicola* inhibited the hippocampal inflammation in OVX mice. **(A)** Immunofluorescence of microglia marker Iba1 of DG area and the quantification of the number and size of Iba1 positive cells. Magnification 400 ×. Scale bar = 20 μm. **(B)** The ratio of COX-2, TLR4, Myd88 to β-tubulin and p-JNK to JNK in hippocampus. **(C)** IL-6 mRNA level in hippocampus. **(D)** IL-8 mRNA level in hippocampus. **(E)** MCP-1 mRNA level in hippocampus. **(F)** TNF-α mRNA level in hippocampus. Data are presented as means ± SEM (*n* = 3–4/group). ^*^p < 0.05, ^**^p < 0.01, ^***^p < 0.001.

The increase in the level of hippocampal inflammatory is mainly attributed to microglial activation; therefore, the expression of microglia marker Iba1, microglia activate stimulus molecules COX-2, TLR4, Myd88, JNK, and inflammatory factors IL-6, IL-8, IL-17, MCP-1, and TNF-α in the hippocampus was examined to evaluate the effects of *P. histicola* on central inflammation. Immunofluorescence results showed that the increased content of microglia marker Iba1 of DG area in OVX mice was significantly reduced by *P. histicola* (*p* < 0.05, Figure 6A). Moreover, western blotting showed that *P. histicola* decreased the levels of COX-2, TLR4, and its downstream protein Myd88, JNK (*p* < 0.05, Figure 6B), hence downregulating the levels of inflammatory factors MCP-1, IL-6, IL-8, and TNF-α induced by OVX (*p* < 0.05, Figures 6C–F). However, the expression of IL-17a among the three groups differed insignificantly (Supplementary Figure 5A). These results suggested that *P. histicola* reversed estrogen deficiency-induced microglial activation and inflammatory responses in OVX mice, which might be achieved by inhibiting TLR4/Myd88/JNK MAPK signaling pathway.

### *P. histicola* Increased the Expression of Neurotrophic Factor BDNF Rather Than Monoamine Neurotransmitters

**Figure 7:**
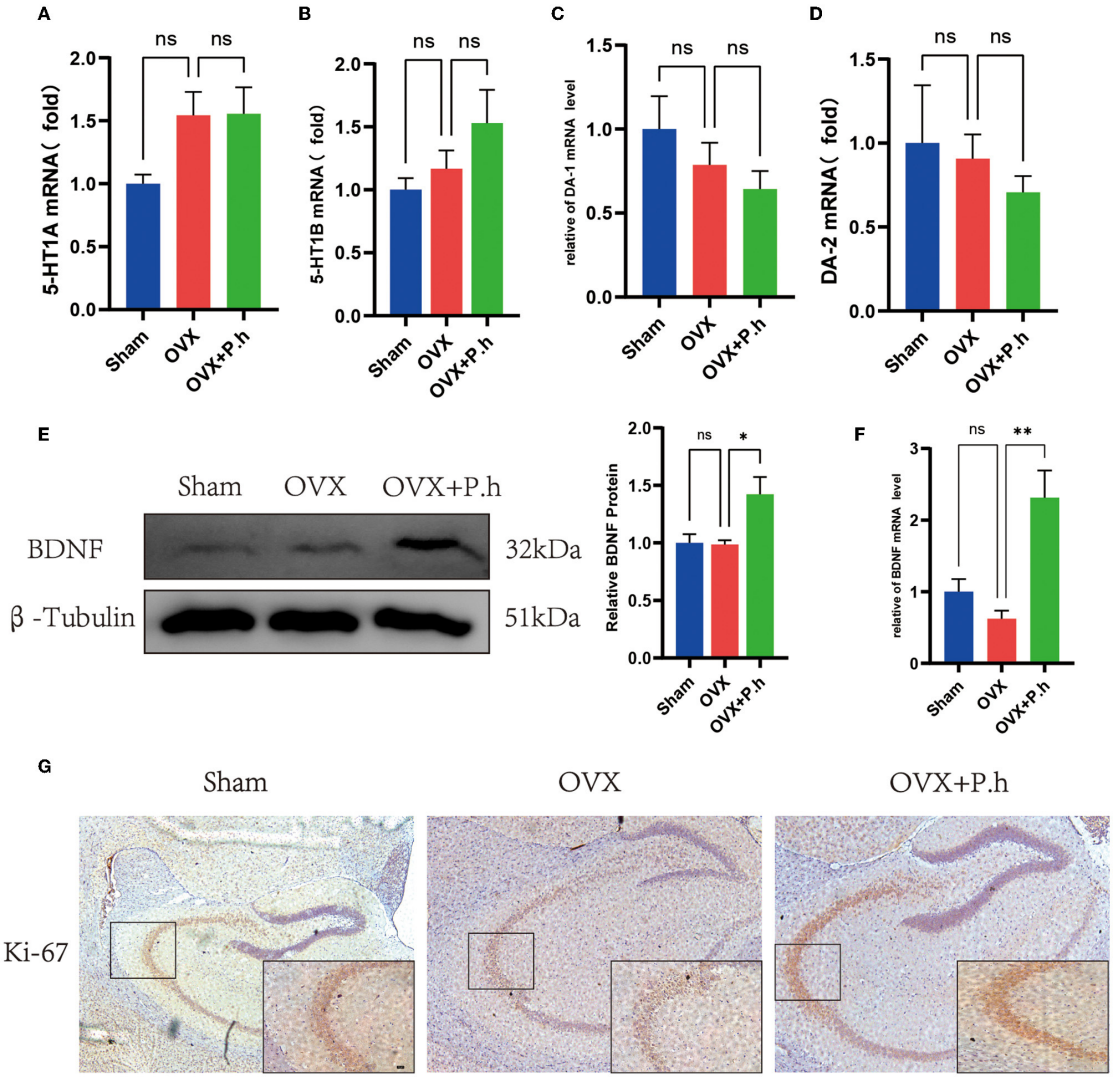
*P. histicola* increased the level of BDNF for nerve proliferation. **(A)** 5-HT1A mRNA level in hippocampus. **(B)** 5-HT1B mRNA level in hippocampus. **(C)** DA-1 mRNA level in hippocampus. **(D)** DA-2 mRNA level in hippocampus. **(E)** BDNF protein level and the ratio of BDNF to β-tubulin in hippocampus. **(F)** BDNF mRNA level in hippocampus. **(G)** Immunohistochemistry of hippocampal proliferation marker Ki-67. Magnification 50 × and 400 ×. Scale bar =20 μm. Data are presented as means ± SEM (*n* = 3–4/group). ^*^p < 0.05, ^**^p < 0.01, ns *p* > 0.05.

Although monoamine neurotransmitters play a critical role in depression, our qRT-PCR results did not show significant changes in receptors 5-HT_1A_, 5-HT_1B_, DA-1, and DA-2 in OVX mice (Figures 7A–D), suggesting that monoamine neurotransmitters may not contribute to the pathogenesis of estrogen deficiency-induced depression. Interestingly, *P. histicola* treatment did not change the expression of 5-HT and DA receptors (Figures 7A–D), suggesting that this pathway was not involved in the action of *P. histicola*. Similarly, the change in the level of BDNF was not found in OVX mice. However, its expression increased significantly after the administration of the *P. histicola* treatment (Figures 7E,F). Further analysis showed that the expression of Ki-67 in the hippocampus of the *P. histicola* group was significantly increased (Figure 7G). Collectively, these data suggest that *P. histicola* increases the level of BDNF to promote neuronal proliferation.

### The Depression of OVX Mice Was Attenuated After Cohousing With *P. histicola*-Treated Mice

**Figure 8:**
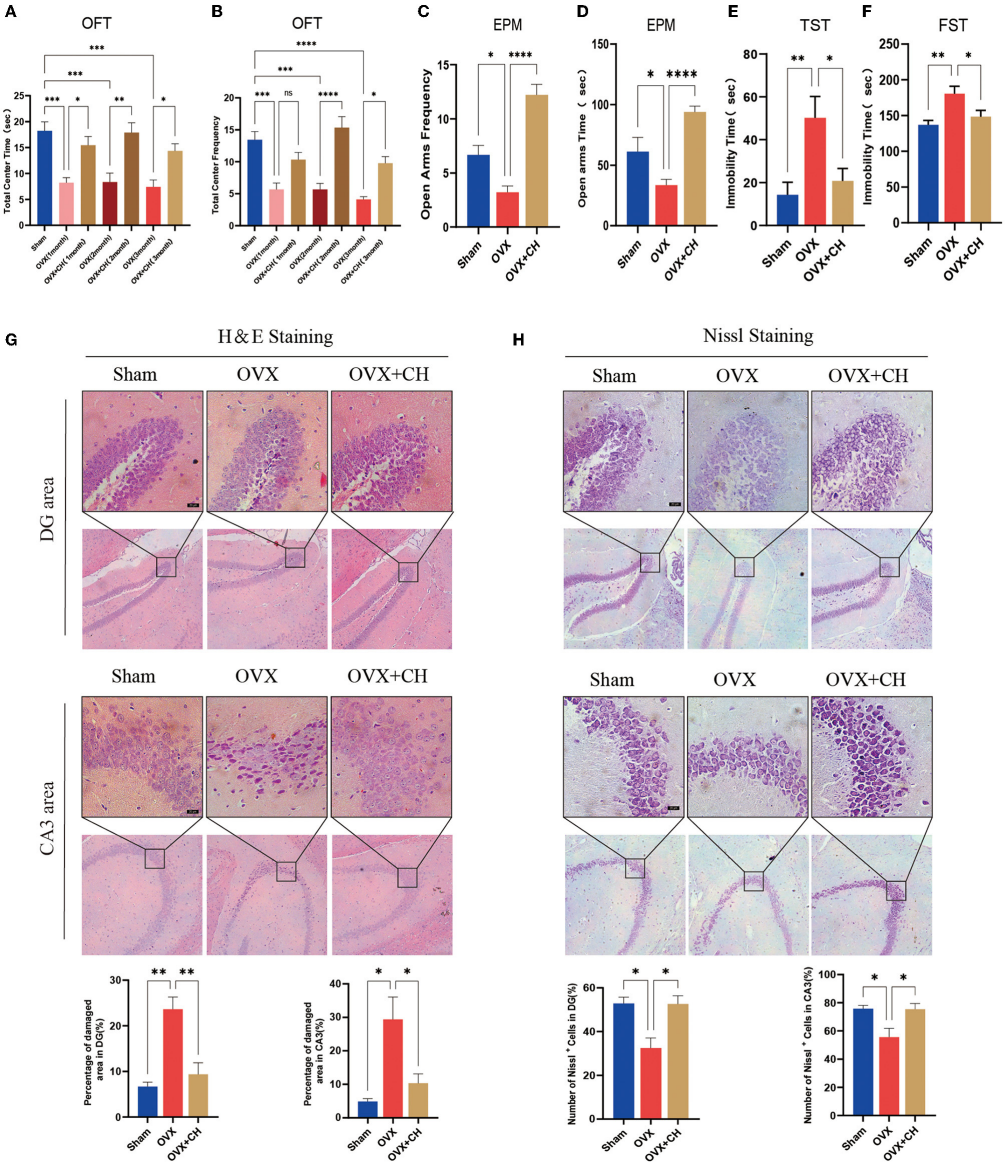
Cohousing with *P. histicola*-treated mice alleviated the depression-like behaviors in OVX. **(A)** Central area time of OFT. **(B)** Transition times of OFT. **(C)** Total time in open arms of EPM. **(D)** Transition times in open arms of EPM. **(E)** Immobility time of TST. **(F)** Immobility time of FST. **(G)** H & E staining and its quantifications. Magnification 100 × and 400 ×. Scale bar = 20 μm. **(H)** Nissl staining and its quantifications. Magnification 100 × and 400 ×. Scale bar = 20 μm. Data are presented as means ± SEM (*n* = 4/group). ^*^p < 0.05, ^**^p < 0.01, ^***^p < 0.001, ^****^p < 0.0001.

To confirm that the antidepressant effect of *P. histicola* was mediated by regulating intestinal microbiota, we randomly selected nine OVX mice and integrated them with *P. histicola-*treated mice. The results of EMP and OFT showed that the time and frequency of entering the open arm and also the central area were significantly increased compared with those in the OVX group (Figures 8A–D). Moreover, the immobility time of OVX mice in TST and FST was significantly reduced in the cohoused mice (*p* < 0.05, Figures 8E,F). As shown by H&E staining, we also found that pyknosis in the nuclei of DG granule cells and hippocampal CA3 pyramidal neurons of OVX mice were attenuated by CH treatment (*p* < 0.05, Figure 8G). Moreover, Nissl staining suggested that CH enhanced protein synthesis in OVX mice (*p* < 0.05, Figure 8H). These data indicated that the antidepressant effect of *P. histicola* was mainly achieved by adjusting the intestinal microbiota.

## Discussion

Estrogen deficiency is considered as an important risk factor for depression (36). In this study, the mice that underwent OVX exhibited significant depression-like behaviors and cognitive impairment, accompanied by intestinal flora disorders. However, following treatment with *P. histicola*, the depression-like behaviors of OVX mice were significantly attenuated. In consistent with the results of *P. histicola*-treated mice, the cohoused OVX mice exhibited the similar antidepressant effects. At the molecular level, the results showed that *P. histicola* treatment reduced central inflammation and upregulated BDNF expression in the hippocampal subregions, which may explain the mechanisms of the antidepressant-like effects of *P. histicola*.

The OVX mice is one typical experimental model for PMD research. Consistent with the previous preclinical studies, our current data revealed that the hippocampal neuronal damage in depression is characterized by apoptosis, which involves the activation of the key proapoptotic caspase-3 and caspase-8 (37, 38) in glial cells and neurons of OVX mice (3). Clinical studies have also confirmed the increased number of apoptotic neurons in hippocampus region in patients with depression (39). Notably, this study found that *P. histicola* significantly reduces the structural damage of neurons and downregulated the caspase-3 and caspase-8 in hippocampal area, suggesting that the antiinjury effect in neurons was achieved by regulating the expression of proapoptotic proteins.

More evidences show that the overproduction of proinflammatory cytokines in the brain results in the neural apoptosis and is central to the development of depression symptoms (40). The increased level of proinflammatory cytokines such as IL-1β, IL-6, and IFN-γ that found in the peripheral circulation and hippocampus of depressive patients (41) has been shown contributing to the development of depression-like behaviors in animal models with estrogen deficiency (42). Microglia, the central nervous system-resident macrophages, are rapidly activated and move toward the site of injury when threatened signals, such as bacterial invasion or signs of injury, are encountered (42–44). Related studies showed that activated microglia acquire neurotoxic and depressive functions owing to the upregulation of proinflammatory cytokines, including IL-6, IL-1β, IL-18, IL-12, IL-23, and TNF-α (45). Our results indicated that the expression of hippocampal proinflammatory factors MCP-1, IL-6, IL-8, and TNF-α was significantly increased as a result of microglial aggregation. These parameters were recovered after the administration of *P. histicola*, suggesting that *P. histicola* intervention reversed the central inflammatory activation in OVX mice.

The pattern recognition receptor toll-like receptors (TLRs), especially TLR4, are critical to initiate the inflammatory response for microglia to promote the occurrence of depression (46, 47) and characterized by increased proinflammatory cytokines TNF-α, IL-1β, IL-6, and IL-12 through the Myd88-dependent pathway (48, 49). Our data show that the level of TLR4 and its downstream protein Myd88 were upregulated in the hippocampus of OVX mice. Moreover, the phosphorylation of key downstream signal protein JNK MAPK was also reduced by *P. histicola* treatment. Previously, in the LPS-injected mice, elevated JNK phosphorylation level is observed in neuroinflammation (50). Salehpour et al. (51) found that near-infrared photobiomodulation combined with coenzyme Q _10_ reduced the level of neuroinflammation in restraint stress mice. Furthermore, the study of Zhang et al. (52) showed that inhibition of JNK reduced the activation of proinflammatory cytokines IL1-β, TNF-α and ameliorated depressive-like behaviors. In contrast, activation of JNK induced hippocampal inflammation and subsequent depression-like behaviors in rats (53). Our results indicate that *P. histicola* might decrease inflammation and cellular dysfunction in hippocampus *via* TLR4/Myd88/JNK MAPK axis-dependent signaling.

Recently, increasing study indicated that the intestinal flora involve in the regulation of the inflammatory response (54) and participate in the development of depression progress (54, 55), which suggest that to clarify the changes in the intestinal flora in OVX mice is important. Although there are few reports on the association between PMD and gut microbes, several animal studies have identified intestinal dysbiosis in OVX mice with estrogen deficiency-induced depression (34, 56, 57). Consistently, our 16S rRNA results suggest that OVX mice had significant intestinal microbiota changes, which were recovered through *P. histicola* treatment. To confirm the connection between intestinal microbiota regulation and the antidepressant effects of *P. histicola*, we selected a group of OVX and mixed them with *P. histicola-*treated mice. In line with this expectation, the behavioral and pathological changes in the mice were significantly improved after cohousing treatment, suggesting that the intestinal microbiota was the key for *P. histicola* against depression. Furthermore, we identified a significant reduction in *Alloprevotella, Allobaculum, Lactobacillus*, and *Akkermansia* and an increase in *Muribaculaceae* in OVX mice. The functions of *Alloprevotella, Allobaculum*, and *Muribaculaceae* are not yet clear, so whether they play a role in the development of depression remains to be further explored. *Lactobacillus*, a genus of gram-positive bacteria, is a constituent of many commonly used probiotics and a significant contributing factor of depression. Ma et al. confirmed that chronic treatment with *Lactobacillus rhamnos* mitigated depression by upregulating GABA in the hippocampus (58). *Lactobacillus helveticus* was reported to prevent depression in humans by decreasing the serum kynurenine-to-tryptophan ratio (59). Furthermore, *Lactobacillus plantarum 299v, Lactobacillus mucosae*, and *Lactobacillus reuteri NK33* were also found to inhibit the occurrence of depression (60–62). *Akkermansia muciniphila, which* is a member of *Akkermansia* genus, has been found to have therapeutic effects in metabolic diseases such as obesity and diabetes (63). The decrease in *Akkermansia muciniphila* was found to destroy intestinal barrier function, cause gut leakage, and activate inflammation and metabolic disorders (64). In addition to being associated with metabolic diseases, *Akkermansia muciniphila* was also reported to be vital in combating psychiatric disorders, owing to the fact that its supplementation increased the level of the antiinflammatory factor IL-10, and recovered the decline of neurotransmitter synthesis caused by the decrease in Nissl bodies (65). Our experiments showed that the quantities of these two probiotics significantly recovered after the administration of the *P. histicola* treatment, indicating that the changes in *Lactobacillus* and *Akkermansia* are significant contributing factors of PMD.

Our KEGG metabolic pathway analysis showed that *P. histicola* altered functional genes in the microbial community, mainly related to the metabolism of terpenoids and polyketides, immune system, tight junction, cell growth and death, and biosynthesis of other secondary metabolites. Previous related studies showed that the gut microbiota, especially *Clostridiales, Lachnospiraceae*, and fermented terpenoids, such as crocin-I and lycopene, produce antiinflammatory effects against depression (21, 66). Interestingly, we found that during the secondary metabolites, the tryptophan metabolite 3-[(4-chlorophenyl) selanyl]-1-methyl-1H-indole, which has been shown to significantly regulate immune, inflammatory, and nervous system diseases (67), was increased by the *P. histicola* treatment. Previous studies revealed that indoles, the microbial metabolites of tryptophan, interacted with pregnane X receptors to improve mucosal homeostasis and barrier function (68). Moreover, indoles act as hydroxyl radical scavengers, neuroprotectants, and human aryl hydrocarbon receptor selective agonists attenuating inflammation (69). Since our results of KEGG metabolic pathways indicated that *P. histicola* might regulate indoles production, this direction is worth to further study in the subsequent experiments.

Previous studies suggest that systemic inflammation plays a pivotal role in depression (42). Moreover, patients with PMD exhibit aberrant immune systems with elevated levels of proinflammatory cytokines, IL-1β and TNF-α, suggesting the pathological involvement of peripheral inflammation in PMD (70). Interestingly, some studies showed that antibiotic-treated mice manifest typical depressive symptoms with elevated inflammatory cytokines in serum resulting from gut leakage induced by dysbiosis (71), indicating that inflammation is a significant link between intestinal flora and depression. Therefore, we speculated that the antidepressant effect of *P. histicola* was achieved by inhibiting the inflammatory response resulting from intestinal leakage. Our published manuscript revealed that *P. histicola* alleviates the increased levels of TNF-α and IL-1β in the circulation of OVX mice (1). Similarly, the data reported herein demonstrate reduced levels of intestinal IL-8, TNF-α, and MCP-1 in *P. histicola*-treated OVX mice, with the upregulation of tight junction proteins ZO-1, occludin, claudin-1, and MUC-2, indicating that *P. histicola* restores the function of the intestinal mucosal barrier. The changes in proinflammatory cytokines and tight junction proteins mainly occurred in the ileum and colon, which might be related to the higher bacterial abundance in these segments of the gastrointestinal tract.

Notably, the probiotic *P. histicola* we chose has been shown the antiinflammatory effects through intestinal microbiota regulation in previous studies. Moreover, *P. histicola* inhibits the activation of microglia and astrocytes, reduces the proinflammatory immune response of the cortex, and reduces the production of inflammatory factors IL-17 and IFN-γ in mice with autoimmune encephalomyelitis (72). Our experiment did not find changes in the level of IL-17 in these animals; however, we found that *P. histicola* significantly decreases the levels of inflammatory factors MCP-1, IL-6, IL-8, and TNF-α in microglia, which were activated by the TLR4/Myd88/JNK MAPK pathway. Recently, bile acids were also reported to be deeply involved in the gut–brain axis in different neural diseases, even chenodeoxycholic acid and tauroursodeoxycholic acid as treatments for Alzheimer's disease (73) and depression, respectively (74). Bhargava P et al. found that tauroursodeoxycholic acid could act on the receptors farnesoid X receptor and G protein-coupled bile acid receptor of microglia to downregulate the expression of nitric oxide synthase 2, IL-1α and TNF-α against inflammation (75). Accordingly, we will design experiments to investigate the effects of *P. histicola* on bile acids metabolism for the underlying mechanical study.

Besides inflammation, brain monoamine interruption is a classic pathogenesis with the changes in DA, 5-HT, and their receptors (76, 77). However, our study did not find any variation in monoamine neurotransmitter receptors after OVX, indicating that monoamine neurotransmitters are not involved in the pathological mechanism of estrogen deficiency depression. Additionally, intragastric administration of *P. histicola* exerted no obvious effects on the levels of neurotransmitter receptors (5-HT_1A_, 5-HT_1B_, DA-1, and DA-2) in the hippocampus. The decline of hippocampal BDNF level caused by inflammatory activation is another pathological basis for depression. In humans, low levels of BDNF are associated with a higher risk and inferior prognosis of neurological diseases (78). Zhao et al. (79) reported that LPS-injected mice exhibit depressive-like behaviors due to a reduction in BDNF induced by inflammatory. Moreover, the infusion of BDNF into the hippocampus has been shown to improve depression-like behaviors in rats (80), suggesting that BDNF has antidepressant effects *in vivo*. Inconsistent with these studies, we did not find a significant change in the expression of hippocampal BDNF after OVX, but *P. histicola* significantly upregulate the level of BDNF, whereas the nerve proliferation marker Ki-67 is also highly expressed after treatment with *P. histicola*, indicating that *P. histicola* promoted nerve proliferation to alleviate depression by upregulating BDNF.

## Conclusions

**Figure 9:**
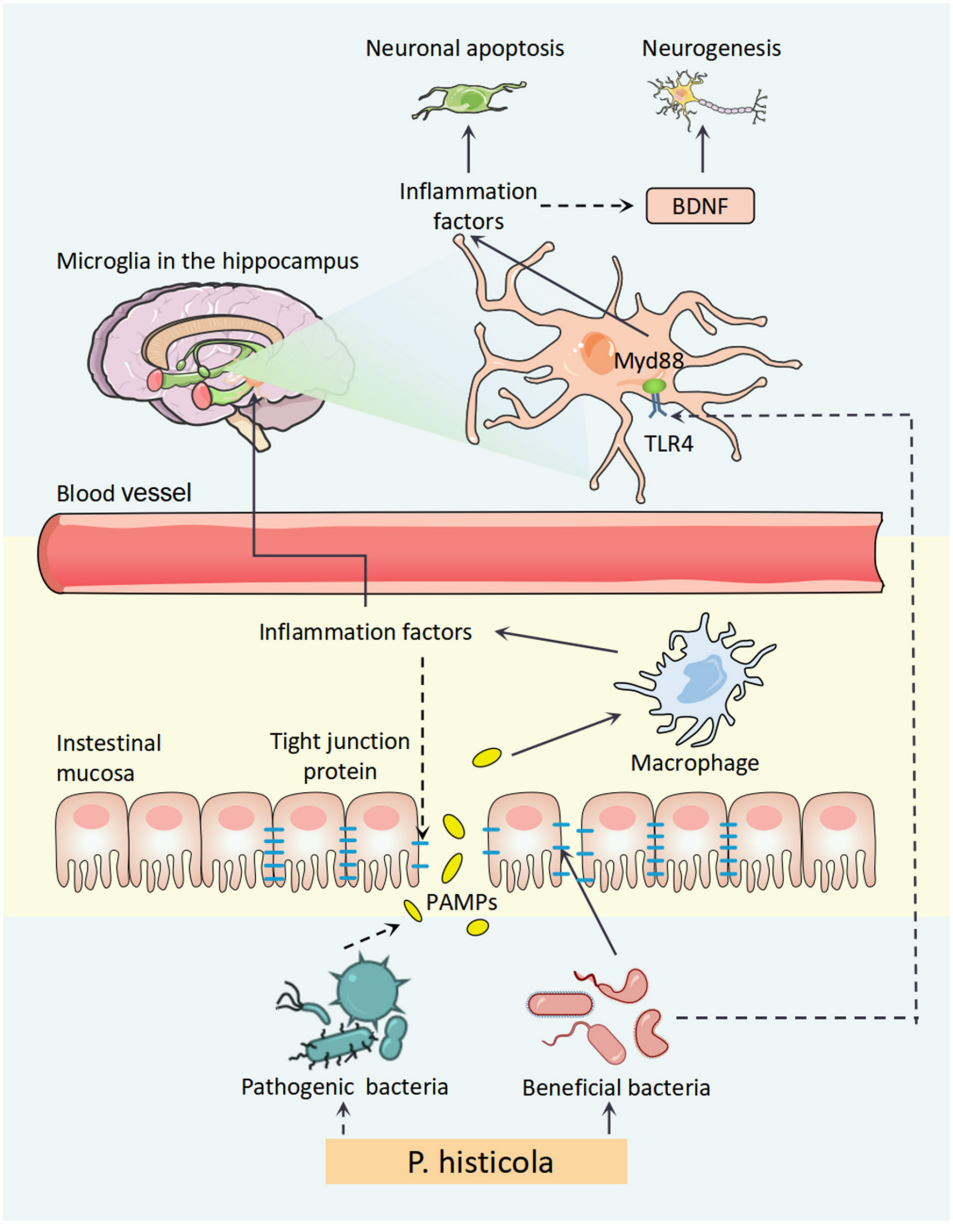
A schematic diagram for the mechanism of *P. histicola* prevention of estrogen deficiency-induced depression *via* the microbiota–brain axis. *P. histicola* treatment exerted a neuroprotective effect through downregulating peripheral and central inflammation, the link of microbiota–gut–brain axis, in estrogen deficiency-induced depression model mice. *P. histicola, Prevotella histicola*; PAMPs, pathogen-associated molecular pattern; TLR4, toll-like receptors 4; Myd88, myeloid differentiation factor 88; BDNF, brain-derived neurotrophic factor.

In summary, our findings elucidated the antidepressant effects of *P. histicola* in OVX mice *via* the regulation of disorder microbiota to attenuate central inflammation, which might be involved in TLR4/Myd88/JNK MAPK pathway (Figure 9) and further upregulate BDNF expression for hippocampal neurogenesis. Finally, our research might provide a novel treatment method for PMD.

## Data Availability Statement

The original contributions presented in the study are publicly available. This data can be found here: https://www.jianguoyun.com/p/DRSDBZsQh8LpCRiQ55EE.

## Ethics Statement

The animal study was reviewed and approved by Laboratory Animal Center of Wenzhou Medical University. Written informed consent was obtained from the owners for the participation of their animals in this study.

## Author Contributions

FW, LC, and CW were responsible for the study concept and design. FH, XL, SH, SW, and SL collected the data. FH, KW, SH, DS, and QL analyzed and interpreted the data. XL, SH, KJ, SZ, and ZW drafted the manuscript. FW critically revised the manuscript for important intellectual content and supervised the study. All authors read and approved the final manuscript.

## Funding

This work was supported by the Basic Public Welfare Research Project of Zhejiang Natural Science Foundation of China (LGF18H090001, LGF22H090028, and LGF22H060014), Wenzhou Basic Science and Technology Cooperation Project (H20180002), Zhejiang Province Medicine and Health Science and Technology Plan (2019KY446), the Natural Science Funds for Distinguished Young Scholar of Zhejiang (LR20H090001), and National Natural Science Foundation of China (82171527).

## Conflict of Interest

The authors declare that the research was conducted in the absence of any commercial or financial relationships that could be construed as a potential conflict of interest.

## Publisher's Note

All claims expressed in this article are solely those of the authors and do not necessarily represent those of their affiliated organizations, or those of the publisher, the editors and the reviewers. Any product that may be evaluated in this article, or claim that may be made by its manufacturer, is not guaranteed or endorsed by the publisher.

## Supplementary Material

Supplemental Figure 1 The mice continued to gain weight after OVX, and *P. histicola* had no significant effect on it. **(A)** The wight of uterus. **(B)** The weight of mice. ^****^*p* < 0.0001.

Supplemental Figure 2 *P. histicola* significantly increased the total distance and mean speed of OVX mice in OFT **(A,B)**. **(A)** Total distance and **(B)** mean speed of OVX mice in OFT. ^*ns*^*p* > 0.05, ^*^*p* < 0.05.

Supplemental Figure 3 OVX and *P. histicola* did not influence intestinal bacterial diversity **(A,B)**. **(A)** Alpha diversity index ACE and **(B)** Chao. ^*ns*^*p* > 0.05.

Supplemental Figure 4 OVX and *P. histicola did* not destroy intestinal mucosa **(A,E,H)**. Magnification 200 × and 400 ×. Scale bar = 20 μm.

Supplemental Figure 5 OVX and *P. histicola* did not regulate the level of hippocampal IL-17a. **(A)** The mRNA expression of hippocampal IL-17a. ^*ns*^*p* > 0.05.
