# Supplementary material for: Prevotella histicola Mitigated Estrogen Deficiency-Induced Depression via Gut Microbiota-Dependent Modulation of Inflammation in Ovariectomized Mice
Source: Front Nutr. 2022 Jan 26;8:805465. doi: 10.3389/fnut.2021.805465 (PMC8826649; doi:10.3389/fnut.2021.805465)
Supplement: Supplementary file 1 [file Table_1.DOCX]

**Supplemental Table 1.** Components of modified PYG medium.

| Components | Contents (/L) |
| --- | --- |
| Trypticase peptone | 5.00 g |
| Peptone | 5.00 g |
| Yeast extract | 10.00 g |
| Beef extract | 5.00 g |
| Glucose | 5.00 g |
| K_2_HPO_4_ | 2.00 g |
| Tween 80 | 1.00 ml |
| Cysteine-HCI x H_2_O | 0.50 g |
| Resazurin | 1.00 mg |
| Salt solution (see below) | 40.00 ml |
| Distilled water | 950.00 ml |
| Haemin solution (see below) | 10.00 ml |
| Vitamin K_1_ solution (see below) | 0.20 ml |
| **Salt solution:** |  |
| CaCI_2_ x 2 H_2_O | 0.25 g |
| MgSO_2_ x 7 H_2_O | 0.50 g |
| K_2_HPO_4_ | 1.00 g |
| KH_2_PO_4_ | 1.00 g |
| NaHCO_3_ | 10.00 g |
| NaCl | 2.00 g |
| Distilled water | 1000 ml |
| **Haemin solution:** |  |
| Haemin | 50 mg |
| Distilled water | 100 ml |
| **Vitamin K_1_ solution:** |  |
| Vitamin K_1_ | 0.1 ml |
| 95% ethanol | 20 ml |
