# Supplementary material for: Prevotella histicola Mitigated Estrogen Deficiency-Induced Depression via Gut Microbiota-Dependent Modulation of Inflammation in Ovariectomized Mice
Source: Front Nutr. 2022 Jan 26;8:805465. doi: 10.3389/fnut.2021.805465 (PMC8826649; doi:10.3389/fnut.2021.805465)
Supplement: Supplementary file 2 [file Table_2.DOCX]

**Supplemental Table 2.** qRT-PCR primer sequences for the targeted mouse genes.

| **Primers** | **Forward Sequence** | **Reverse Sequence** |
| --- | --- | --- |
| IL-6 | TCACAGAAGGAGTGGCTAAGGACC | ACGCACTAGGTTTGCCGAGTAGAT |
| IL-8 | ATGACTTCCAAGCTGGCCGTGGCT | TCTCAGCCCTCTTCAAAAACTTCTC |
| TNF-α | CTTGTTGCCTCCTCTTTTGCTTA | CTTTATTTCTCTCAATGACCCGTAG |
| MCP-1 | ACCTGCTGCTACTCATTCACC | ATTCCTTCTTGGGGTCAGCA |
| Caspase-3 | ATGGGAGCAAGTCAGTGGAC | GTCCACATCCGTACCAGAGC |
| Caspase-8 | GCTTCGAGCAACAGAACCAC | ACGCCAGTCAGGATGCTAAG |
| BDNF | GTTCGAGAGGTCTGACGACG | TGTTTGCGGCATCCAGGTAA |
| ZO-1 | AACCCGAAACTGATGCTGTG | CCCTTGGAATGTATGTGGAGAG |
| Occludin | CCCAGATTAGAGTCCAAAGTCAGT | CGGAAACCTTAGAGAGATGCC |
| Claudin-1 | GGAGTCAGTGTTTCAGCCTATGGT | GAAGGGTTCATGCCTCTCATCT |
| MUC2 | TGCGTGTTCCTGATGCTGAT | TCTCGTGGCGCACAATAAGT |
| 5-HT1A | CAGGGCAACAACACCACAAC | CCCCAGCAAAAGAGAGGTGA |
| 5-HT1B | CACCCTTCTTCTGGCGTCAA | GAGAGCGGGCTTCCACATAG |
| DA-1 | AGGTTGAGCAGGACATACGC | TTGCTTCTGGGCAATCCTGT |
| DA-2 | GTCTCGTTCTACGTGCCCTT | CTGCTACGCTTGGTGTTGAC |
| β-actin | CTAAGGCCAACCGTGAAAAG | GGTACGACCAGAGGCATACA |
