# Supplementary material for: Prevotella histicola Mitigated Estrogen Deficiency-Induced Depression via Gut Microbiota-Dependent Modulation of Inflammation in Ovariectomized Mice
Source: Front Nutr. 2022 Jan 26;8:805465. doi: 10.3389/fnut.2021.805465 (PMC8826649; doi:10.3389/fnut.2021.805465)
Supplement: Supplementary file 3 [file Data_Sheet_1.docx]

**Supplemental Figure 1：The mice continued to gain weight after OVX, and *P. histicola* had no significant effect on it. A.** The wight of uterus. **B.** The weight of mice. *****p* <0.0001.

**Supplemental Figure 2: *P. histicola* significantly increased the total distance and mean speed of OVX mice in OFT** **A & B.** Total distance (A) and mean speed (B) of OVX mice in OFT. ^ns^*p* >0.05, **p* <0.05.

**Supplemental Figure 3：OVX and *P. histicola* did not influence intestinal bacterial diversity.** **A & B.** Alpha diversity index ACE (A) and Chao (B). ^ns^*p* >0.05.

**Supplemental Figure 4：OVX and *P. histicola* did not destroy intestinal mucosa.** **A.** H & E. Magnification 200× and 400×. Scale bar = 20 μm.

**Supplemental Figure 5: OVX and *P. histicola* did not regulate the level of hippocampal IL-17a. A.** The mRNA expression of hippocampal IL-17a. ^ns^*p* >0.05.
