# Supplementary figures and images for: Prevotella histicola Mitigated Estrogen Deficiency-Induced Depression via Gut Microbiota-Dependent Modulation of Inflammation in Ovariectomized Mice
Source: Front Nutr. 2022 Jan 26;8:805465. doi: 10.3389/fnut.2021.805465 (PMC8826649; doi:10.3389/fnut.2021.805465)

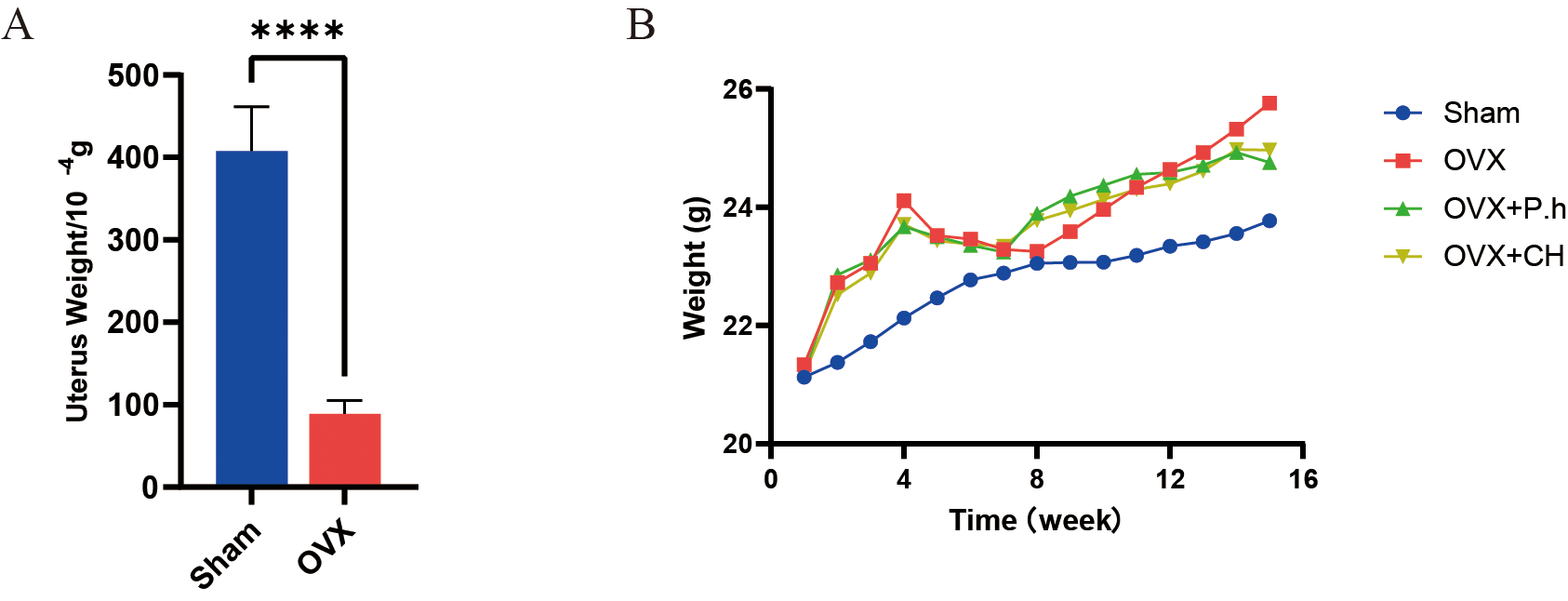

Supplement: Supplemental Figure 1 — The mice continued to gain weight after OVX, and P. histicola had no significant effect on it. (A) The wight of uterus. (B) The weight of mice. ****p < 0.0001. [file Image_1.TIF]

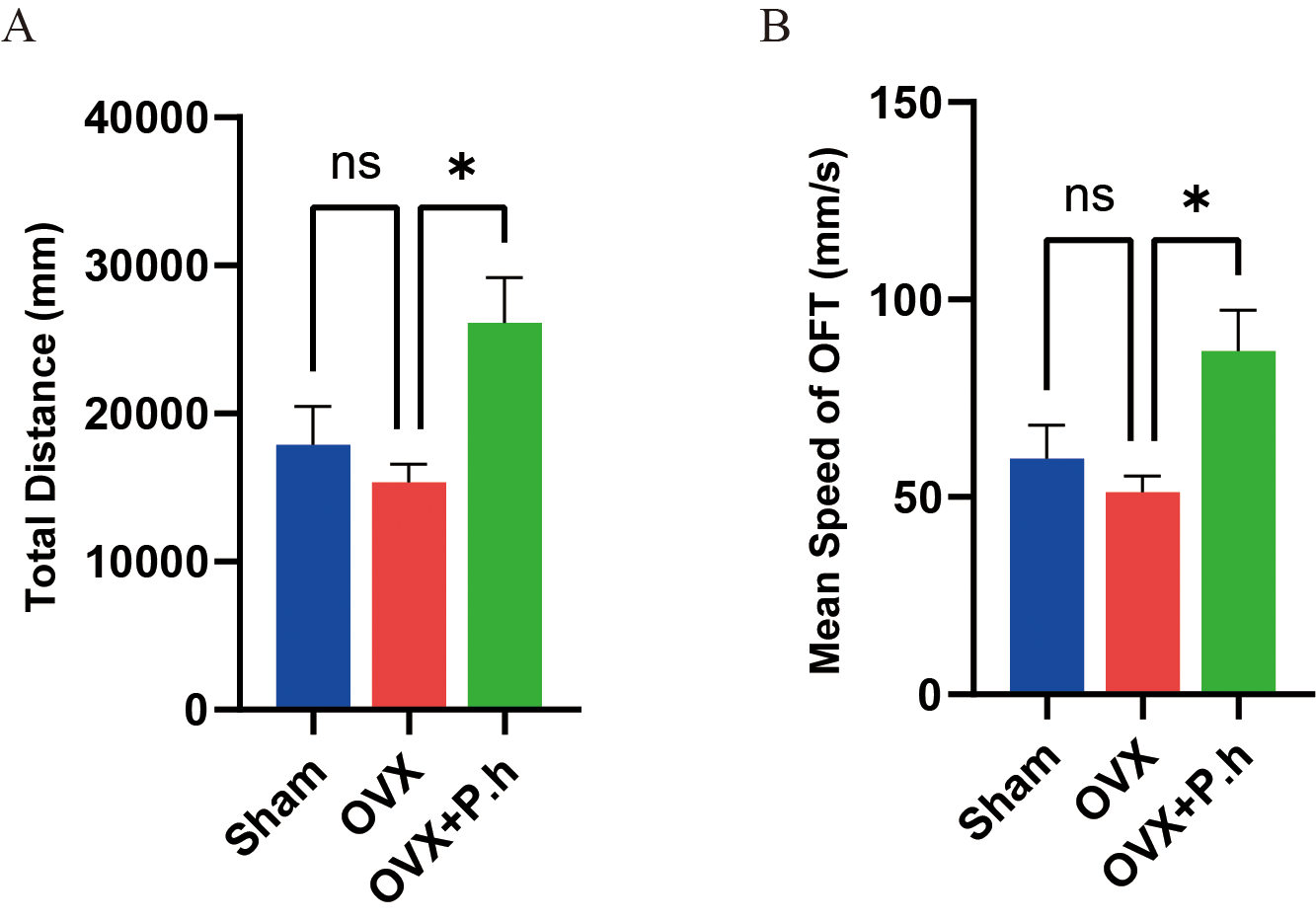

Supplement: Supplemental Figure 2 — P. histicola significantly increased the total distance and mean speed of OVX mice in OFT (A,B). (A) Total distance and (B) mean speed of OVX mice in OFT. nsp > 0.05, *p < 0.05. [file Image_2.TIF]

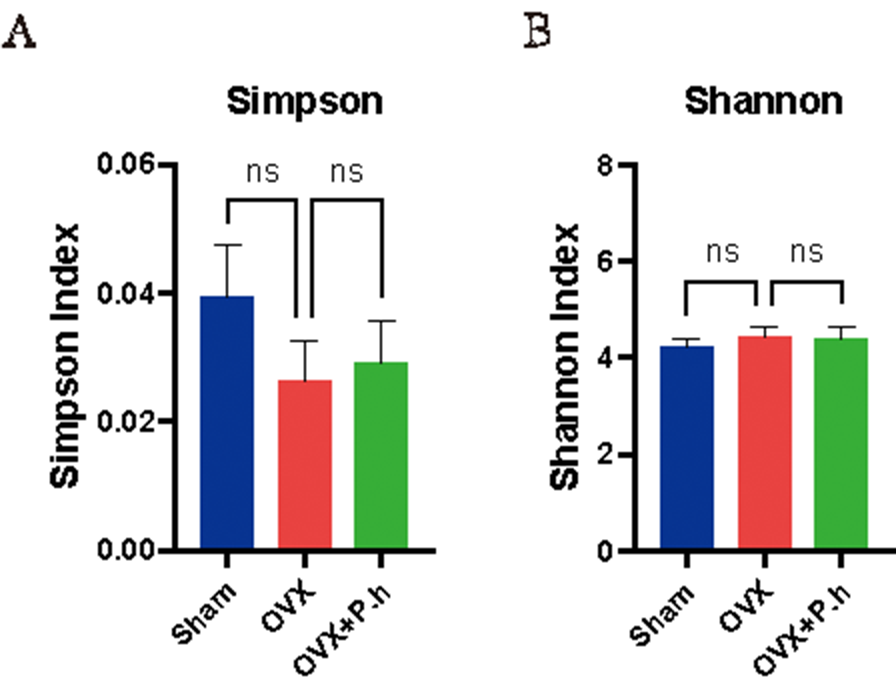

Supplement: Supplemental Figure 3 — OVX and P. histicola did not influence intestinal bacterial diversity (A,B). (A) Alpha diversity index ACE and (B) Chao. nsp > 0.05. [file Image_3.TIF]

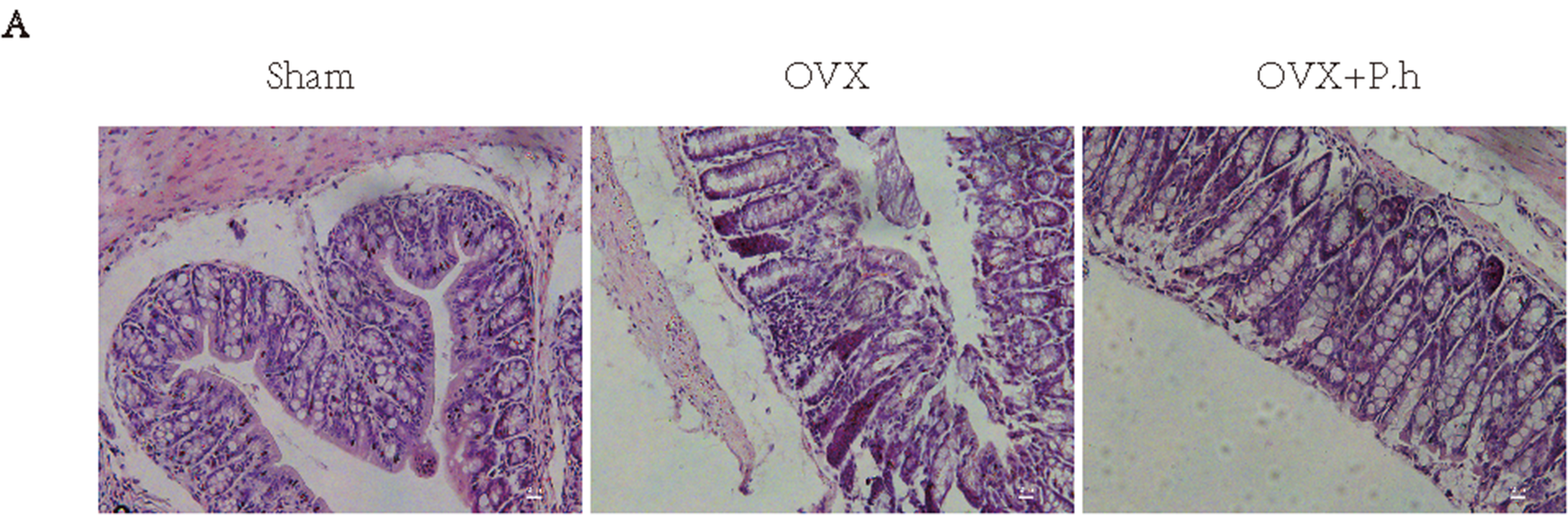

Supplement: Supplemental Figure 4 — OVX and P. histicola did not destroy intestinal mucosa (A,E,H). Magnification 200 × and 400 ×. Scale bar = 20 μm. [file Image_4.TIF]

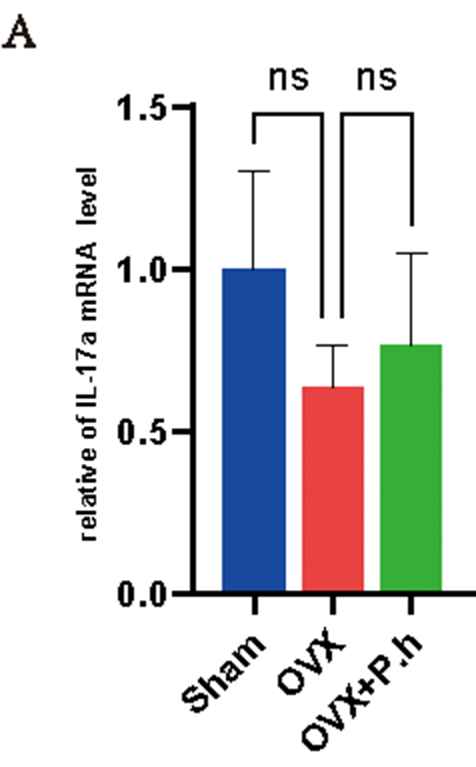

Supplement: Supplemental Figure 5 — OVX and P. histicola did not regulate the level of hippocampal IL-17a. (A) The mRNA expression of hippocampal IL-17a. nsp > 0.05. [file Image_5.TIF]
